# Effects of Diabetic Complications on Health-Related Quality of Life Impairment in Vietnamese Patients with Type 2 Diabetes

**DOI:** 10.1155/2020/4360804

**Published:** 2020-01-23

**Authors:** Tuyen Ba Pham, Trung Thanh Nguyen, Huyen Thi Truong, Chin Huu Trinh, Ha Ngoc Thi Du, Tam Thi Ngo, Long Hoang Nguyen

**Affiliations:** ^1^Traditional Medicine Hospital, Ministry of Public Security, Hanoi 100000, Vietnam; ^2^VNU School of Medicine and Pharmacy, Vietnam National University, Hanoi 100000, Vietnam; ^3^Faculty of Health Sciences, Thang Long University, Hanoi 100000, Vietnam

## Abstract

Complications of type 2 diabetes mellitus (T2DM) adversely influence patients' health-related quality of life (HRQOL). This study is aimed at examining HRQOL of T2DM patients, as well as the effects of diabetic complications and comorbidities on HRQOL in this population. This was a hospital-based cross-sectional study on 214 T2DM patients in Hanoi, Vietnam. Short-form 12 version 2 (SF-12v2) and EuroQOL-5 Dimensions-5 Levels (EQ-5D-5L) were employed to measure the HRQOL. The median physical component summary score (PCS), mental component summary score (MCS), and EQ-5D index were 45.6, 56.3, and 0.94, respectively. Having at least one diabetic complication was associated with the reduction of SF-12 scores in social functioning (Diff. = −5.69, 95%CI = −9.24; -2.13), role emotional (Diff. = −1.81, 95%CI = −3.12; -0.51), and MCS (Diff. = −2.55, 95%CI = −5.01; -0.1). Significant decrement of physical functioning, role physical, social functioning, role emotional, and MCS was found in patients having diabetic heart diseases compared to those without diabetic complications. The study revealed that HRQOL of Vietnamese patients with diabetic complications was moderately low, especially in social and mental health perspectives. Strategies to prevent the onset of diabetic complications should be developed as a priority in diabetes management.

## 1. Introduction

Type 2 diabetes mellitus (T2DM) is increasing at an alarming rate and has been recognized as one of the leading causes of death and disabilities worldwide [1]. Uncontrolled and untreated T2DM leads to serious complications, such as promoting the coagulation of blood, diabetic retinopathy, hypertension, chronic kidney disease, or foot ulceration [2], and significantly reduces patients' productivity and life expectancy [3]. Recent estimates indicate that in 2017, more than 400 million people are living with diabetes [4], and this figure was predicted to increase by nearly 200 million in 2035 [5]. Moreover, in 2017, 3.2 million deaths are attributable to diabetes in people aged 60-99 years [2]. These figures raise an urgent need to further assess the impact of diabetes on the lives of patients.

Health-related quality of life (HRQOL) is defined as “an individual's perception of the extent to how diseases, disability, or disorder affect physical, emotional, and social status” [6, 7]. Enhancing the quality of life is the ultimate goal of treating T2DM even though clinical indicators provide a good estimate of controlling this illness [8]. People who live with T2DM suffer a heavy burden of treatment and diabetic complications, which alleviate their HRQOL remarkably compared to those without the diseases [9]. Prior literature indicates that inability to perform physical function due to complications and feeling anxious or depressed because of high glycaemic is the cause of reduced HRQOL in diabetic patients [9–14]. Understanding the effects of diabetic complications on HRQOL is thus necessary to develop more appropriate treatment guidelines for diabetic patients [15].

Vietnam is among the low-middle-income countries with the highest burden of diabetes [16, 17]. In 2017, it was an estimate of about 5.5% of adults living with diabetes [4]. The significant rise in the incidence of T2DM in Vietnam also reaches an alarming level when it has doubled in 10 years from 2.7% in 2002 to 5.4% in 2010 [18, 19]. Two previous studies indicate the significant impairment of HRQOL in Vietnamese elderly diabetic patients, as well as correlations between HRQOL and comorbidity, duration of disease, frequency of self-monitoring, and burden of finance [20, 21]. However, studies on the associations between diabetic complications and HRQOL impairment in diabetic patients are lacking. Therefore, this study is aimed at examining HRQOL of T2DM patients, as well as the effects of diabetic complications and comorbidities on HRQOL in this population.

## 2. Materials and Methods

### 2.1. Study Design and Sampling Technique

A cross-sectional study was performed in July 2019 in the Outpatient Department of Traditional Medicine Hospital of the Ministry of Public Security, Hanoi, Vietnam. This hospital manages more than two thousand patients with diabetes. Patients were not only police officers but also the general population residing in Hanoi, Vietnam. All participants of the study were diagnosed with T2DM according to the official guideline of Vietnam's Ministry of Health. They were registered for chronic disease management and treated as outpatients in the hospital. They were excluded if having central nervous system (CNS) diseases such as schizophrenia, or psychosis, or being unable to communicate due to congenital dumbness of deafness based on the diagnosis of physicians. Patients who were inpatients were also not able to participate in this survey.

We calculated the sample size to estimate the difference between two population means [22]. With two-tailed significance level *α* = 0.05, absolute precision required = 0.25, and population standard deviation = 0.74 for PCS-12 between groups with and without diabetic complications (which was found in the previous study in China [23]), the sample size was 68 patients per group. We decided to recruit patients with the ratio between patients with complications (cases) and patients without complications (controls) (1 : 2), resulting in 204 patients being required for the final sample size (68 cases and 136 controls). A simple random sampling technique was applied to recruit patients. After listing all eligible patients with the support of the medical staff, we used a computer software to select randomly a total of 250 patients (100 patients with complications and 150 patients without complications) for preventing refusal, of which 214 patients (response rate 85.6%: 83 cases and 131 controls) agreed to participate in the survey.

After inviting them to be enrolled in the study, patients were briefly explained about the aim of the study and asked if they wanted to participate. If the patient refused for personal reasons, they were not included in the study without any influences on their current treatment. Patients agreeing to participate were invited to go to a private room in the hospital to ensure confidentiality. The Institutional Review Board of School of Medicine and Pharmacy, Vietnam National University, considered and approved all protocols and materials. Written or verbal informed consent forms were obtained from the participants.

### 2.2. Data Measurement

Data collection was performed by six undergraduate medical students at the School of Medicine and Pharmacy, Vietnam National University and Hanoi Medical University. These students were trained intensively to acquire essential communication skills with patients and were proficient in investigating and exploiting information from participants. Moreover, they were trained about measuring patients' blood pressure before conducting the data collection. The questionnaire was piloted in five patients and revised and approved by the research team and leader of the hospital.

In this study, we use the SF-12v2 instrument to measure the HRQOL of T2DM patients [22]. This tool is useful in evaluating the relationship between quality of life and other factors such as complications, age, occupation, gender, and type of treatments among diabetic patients [7, 20, 24]. This tool contains 12 items used to assess eight domains: general health, physical functioning, social functioning, role physical, role emotional, body pain, vitality, and mental health [5]. The score ranges from 0 to 100, where a higher score means better HRQOL. We also calculated the Physical Component Score-12 (PCS-12) and Mental Component Score-12 (MCS-12) based on the responses of participants and the user's manual [24]. Moreover, we employed the EuroQOL-5 Dimensions-5 Levels (EQ-5D-5L) to measure the health utility of T2DM patients. The health utility index was calculated from five items of this tool by using the Vietnam crosswalk value set, with the score ranging from -0.566 to 1 [25]. This instrument has been used in previous studies in Vietnam [26–31]. Cronbach's alpha values for SF-12v2 and EQ-5D-5L were 0.77 and 0.61, respectively.

We also extracted some clinical data from the electronic medical record system of the hospital, including age, sex, duration of diabetes, latest HbA1c (%), and glycaemic level (g/mmol), clinical diagnoses of diabetic complications, and comorbidities. Systolic blood pressure and diastolic blood pressure were measured after resting 10 minutes and before interviewing patients. Diabetic complications in this study included heart diseases (e.g., heart failure, coronary heart diseases, or myocardial infarction), diabetic nephropathy, or diabetic retinopathy.

### 2.3. Statistical Analysis

We used Stata version 14.0 software (StataCorp LP, College Station, Texas, USA) to analyze all data, and the results were statistically significant with a *p* value less than 0.05. Data of the skewness-kurtosis test indicated that age, duration of disease, latest HbA1c, latest glycaemic level, systolic blood pressure, diastolic blood pressure, number of comorbidities, scores of eight SF-12v2 domains, PCS-12, MCS-12, and EQ-5D index had nonnormal distribution. Multivariable regression models were employed to compute the effects of (1) having diabetic complications (Yes/No), (2) having one and two or more diabetic complications (compared with no complications), (3) having diabetic heart diseases (Yes/No), and (4) having diabetic nephropathy (Yes/No) on the SF-12 domains (ordinary least squares (OLS) regression with robust standard errors) and EQ-5D index (Tobit regression). In the literature, several different approaches were proposed to regress the skewed HRQOL [32–34]; OLS regression is one of the commonest regression methods. Furthermore, a previous study found that validity of the OLS model can be comparable with other approaches [35]. Meanwhile, data of the EQ-5D index are censored, which are appropriate for censored models such as Tobit regression. All models were adjusted to clinical characteristics such as age, gender, duration of diabetes, last HbA1c, last glycaemic level, systolic blood pressure, and diastolic blood pressure.

## 3. Results

Table 1 shows the median age was 61.5 (interquartile range (IQR) = 58‐67), with half of the participants aged 60-69 (50.0%). More than half of the participants were female (52.8%). The median duration of disease was 7 years (IQR = 3‐10), whereas the median latest HbA1c was 6.8% (IQR = 6.0‐7.7) and the median latest glycaemic level was 6.8 g/mmol (IQR = 6.1‐8.1). Systolic blood pressure (SBP) and diastolic blood pressure (DBP) had median values of 129 mmHg (IQR = 120‐140) and 80 mmHg (IQR = 70‐80), respectively.

After adjustment for the number of comorbidities, age, sex, duration of disease, HbA1c, glucose, SBP, and DBP, Table 2 shows the SF-12 scores according to the number of diabetic complications, as well as the difference between those with and without complications. Having at least one diabetic complication was associated with the reduction of SF-12 scores in SF (Diff. = −5.69, 95%CI = −9.24; -2.13), RE (Diff. = −1.81, 95%CI = −3.12; -0.51), and MCS-12 (Diff. = −2.55, 95%CI = −5.01; -0.1). The differences in scores of SF and RE in patients with one complication compared to those without diabetic complication were significant. Meanwhile, the significant differences in SF, RE, and MCS-12 between those having two or more diabetic complications and those without complications were observed.

Table 3 shows the significant decrement of PF (Diff. = −3.90, 95%CI = −7.71; -0.09), RP (Diff. = −1.54, 95%CI = −2.94; -0.14), SF (Diff. = −6.16, 95%CI = −10.08; -2.24), RE (Diff. = −2.24, 95%CI = −3.74; -0.74), and MCS-12 (Diff. = −3.10, 95%CI = −5.85; -0.35) in patients having diabetic heart diseases compared to those without diabetic complications. Meanwhile, patients having diabetic nephropathy had significantly lower SF and RE scores than those without diabetic complications. No significant difference of HRQOL was found in patients with and without diabetic retinopathy.

Table 4 reveals that the EQ-5D index was significantly lower among patients having at least one diabetic complication and having one diabetic complication compared to those without any diabetic complications. However, no difference was found between those without diabetic complications and those with diabetic heart disease, diabetic nephropathy, and diabetic retinopathy.

## 4. Discussion

This study informs an insight into the effects of different diabetic complications on the HRQOL of Vietnamese adults with type 2 diabetes. In this study, we found that patients with diabetic complications had significantly lower HRQOL than individuals without complications in PF, RP, SF, RE, and MCS. Heart diseases had the greatest magnitude of HRQOL reduction in physical and social functioning, while nephropathy had the largest adverse effect on social functioning and role emotional. Meanwhile, retinopathy showed a limited relationship with the reduction of HRQOL.

In the diabetes patient sample, the general health domain had the lowest score, followed by physical functioning and bodily pain. Moreover, the PCS and MCS had moderately low scores (median PCS = 45.6 and MCS = 56.3), which are consistent with other studies worldwide such as Singapore [36], China [23], the United Kingdom (UK) [12], and the United States of America (USA) [37]. Besides, we found that health utility measured by using EQ-5D-5L in patients without diabetic complications was relatively similar to the EQ-5D index of the general Vietnamese population (mean = 0.91) [26]. This finding could also be comparable to other studies in developed countries such as Singapore [38], the UK [39], and Norway [40]. Indeed, several studies in other countries found that HRQOL of patients without diabetic complications were not remarkably different from nondiabetic patients [36, 41–44]. Therefore, well-preventing and controlling diabetic complications are an important target to ensure the HRQOL of diabetic patients.

After adjusting to potential clinical confounders, diabetic complications such as nephropathy or retinopathy were associated with the significant decrement of social functioning and emotional role dimensions. We supposed that these illnesses were in mild stage or well-controlled since patients in our study had been managed in the hospital in a long time. Therefore, these diseases did not affect patients' physical status significantly but made patients worry about the influence of these diseases on daily lives as well as a social relationship. Meanwhile, we observed that patients with heart diseases suffered from not only mental problems as similar to two other complications but also the decrease in physical functioning and role physical. Indeed, patients with heart diseases such as heart failure or major/minor vascular illnesses have a significantly higher likelihood of premature death compared to nephropathy or retinopathy [45, 46]. Previous studies informed solid evidence that vascular diseases in diabetic patients were correlated with severe problems in pain/discomfort, mobility, self-care, and usual activities [41, 44, 47].

Unexpectedly, this study only found the significant decreases in social functioning, role emotional, and mental component scores in patients with diabetic complications (both groups with one complication and with two or more complications) compared to those without complications. Results in the EQ-5D index also indicated that patients with one diabetic complication, not those with two or more complications, had a significantly lower score than those without complication. This is different from studies in China and Norway [23, 40], which might be justified by the small sample size and cultural characteristics in our study. However, it implies that prevention to improve the mental health and social perspectives of patients with diabetic complications is critically important to enhance these patients' HRQOL.

Our strengths included the use of validated measures such as the SF-12v2 and EQ-5D index. Moreover, the clinical characteristics of our sample were extracted from electronic medical records, which increase the reliability of our data. However, limitations of this study should be acknowledged. First, this study used the cross-sectional design, which allows drawing conclusions about associations but not causal relationships. This requires further longitudinal study to understand how the onset of diabetic complications impacts the HRQOL. Second, our study was performed in only one hospital in Hanoi, Vietnam, with a small sample size, which might not represent other settings. Third, we did not measure the effects of complications in different stages of illness, which might be an important factor associated with the reduction of HRQOL. Finally, we did not employ a control group, such as a healthy general population to measure the comprehensive burden of diabetic complications.

## 5. Conclusions

The HRQOL of Vietnamese patients with diabetic complications was moderately low, especially in social and mental health perspectives. Strategies to prevent the onset of diabetic complications should be developed as a priority in diabetes management. Health preference utility scores in this study could play as a reference for further health economic evaluations to prevent diabetic complications in Vietnam.

## Figures and Tables

**Table 1:** Sociodemographic characteristics.

Characteristics	Total		With diabetic complications		Without diabetic complications	
	*n*	%	*n*	%	*n*	%
Total	214	100.0	83	100	131	100
Age group						
≤49 years	8	3.7	1	1.2	7	5.3
50-55 years	20	9.3	6	7.2	14	10.7
56-60 years	59	27.6	22	26.5	37	28.2
61-65 years	60	28.0	26	31.3	34	25.9
66-70 years	37	17.3	13	15.7	24	18.3
>70	30	14.0	15	18.1	15	11.4
Gender						
Female	113	52.8	46	55.4	67	51.2
Male	101	47.2	37	44.6	64	48.8
	Median	IQR	Median	IQR	Median	IQR
Age (years)	61.5	58-67	61	57-67	62	60-68
Duration of disease (years)	7	3-10	6	3-10	7	3-10
Latest HbA1c (%)	6.8	6.0-7.7	6.75	6-7.6	6.9	6-7.8
Latest glycaemic level (g/mmol)	6.8	6.1-8.1	7	6.2-8.2	6.7	5.95-8
Systolic blood pressure	129	120-140	130	120-140	120	120-140
Diastolic blood pressure	80	70-80	80	70-80	80	70-80
Number of comorbidities	2	1-3	2	1-2	2	1-3

**Table 2:** HRQOL measured by SF-12 according to the different numbers of complications.

HRQOL	Total (*n* = 214)	Without diabetes complications (*n* = 131)	Number of complications					
			Having diabetes complication (*n* = 83)^b^		Having one diabetic complication (*n* = 67)^b^		Having two or more diabetic complications (*n* = 16)^b^	
			Median (IQR)	Difference (95% CI)^a^	Median (IQR)	Difference (95% CI)^a^	Median (IQR)	Difference (95% CI)^a^
PF	47.9 (39.3-56.5)	47.9 (39.3-56.5)	47.9 (39.3-56.5)	-2.49 (-5.87; 0.89)	47.9 (39.3-56.5)	-1.85 (-5.44; 1.74)	47.9 (39.3-56.5)	-5.82 (-12.98; 1.35)
RP	57.2 (48.0-57.2)	57.2 (52.6-57.2)	52.6 (48-57.2)	-1.43^∗^ (-2.65; -0.21)	52.6 (48-57.2)	-1.21 (-2.51; 0.09)	52.6 (48-57.2)	-2.55 (-5.13; 0.04)
BP	57.4 (37.1-57.4)	57.4 (47.3-57.4)	57.4 (37.1-57.4)	-1.48 (-5.73; 2.76)	57.4 (37.1-57.4)	-1.89 (-6.43; 2.64)	47.3 (26.9-57.4)	0.6 (-8.38; 9.57)
GH	29.6 (29.6-44.7)	29.6 (29.6-44.7)	29.6 (29.6-44.7)	-3.05 (-6.34; 0.25)	29.6 (29.6-44.7)	-2.63 (-6.14; 0.88)	29.6 (29.6-29.6)	-5.21 (-12.21; 1.8)
VT	67.9 (37.7-67.9)	67.9 (37.7-67.9)	57.8 (37.7-67.9)	-0.73 (-5.49; 4.03)	62.8 (37.7-67.9)	-0.9 (-5.99; 4.19)	57.8 (42.7-67.9)	0.13 (-9.89; 10.14)
SF	56.6 (46.5-56.6)	56.6 (56.6-56.6)	56.6 (36.4-56.6)^∗^	-5.69^∗^ (-9.24; -2.13)	56.6 (46.5-56.6)^∗^	-4.83^∗^ (-8.61; -1.06)	41.4 (26.3-56.6)^∗^	-10.0^∗^ (-17.48; -2.52)
RE	56.1 (50.5-56.1)	56.1 (56.1-56.1)	56.1 (50.5-56.1)^∗^	-1.81^∗^ (-3.12; -0.51)	56.1 (50.5-56.1)	-1.49^∗^ (-2.88; -0.1)	56.1 (44.9-56.1)^∗^	-3.44^∗^ (-6.19; -0.69)
MH	58.4 (46.3-64.5)	58.4 (46.3-64.5)	58.4 (46.3-64.5)	-1.9 (-5.49; 1.7)	58.4 (46.3-64.5)	-1.24 (-5.08; 2.6)	49.3 (43.2-64.5)	-5.17 (-12.73; 2.38)
PCS-12	45.6 (38.9-50.5)	46.6 (38.9-50.8)	43.9 (38.1-50)	-1.71 (-4.5; 1.08)	44.7 (38.5-50.6)	-1.53 (-4.51; 1.45)	41.9 (33.1-44.8)	-2.6 (-8.47; 3.26)
MCS-12	56.3 (51.1-62.3)	58.6 (52.1-62.9)	54.7 (49.8-59.9)^∗^	-2.55^∗^ (-5.01; -0.1)	55.1 (50-61.2)^∗^	-2.06 (-4.68; 0.56)	51.7 (45.3-55.8)^∗^	-4.98 (-10.14; 0.17)

Table expressed as median (IQR). BP: bodily pain; GH: general health; MCS: mental component summary score; MH: mental health; PCS: physical component summary score; PF: physical functioning; RE: role emotional; RP: role physical; SF: social functioning; VT: vitality. ^∗^*p* < 0.05. ^a^Multivariate linear regression adjusted for the number of comorbidities, age, sex, duration of disease, HbA1c, glucose, SBP, and DBP. ^b^Compared to the “without complication” group.

**Table 3:** HRQOL measured by SF-12 according to different complications.

HRQOL	Total (*n* = 214)	Without diabetic complications (*n* = 131)	Individual complication					
			Having diabetic heart disease (*n* = 56)^b^		Having diabetic nephropathy (*n* = 18)^b^		Having diabetic retinopathy (*n* = 10)^b^	
			Median (IQR)	Difference (95% CI)^a^	Median (IQR)	Difference (95% CI)^a^	Median (IQR)	Difference (95% CI)^a^
PF	47.9 (39.3-56.5)	47.9 (39.3-56.5)	47.9 (39.3-56.5)	-3.90^∗^ (-7.71; -0.09)	47.9 (39.3-56.5)	-4.43 (-10.39; 1.53)	47.9 (39.3-56.5)	2.55 (-5.63; 10.73)
RP	57.2 (48.0-57.2)	57.2 (52.6-57.2)	52.6 (48-57.2)	-1.54^∗^ (-2.94; -0.14)	52.6 (48-57.2)	-1.86 (-3.93; 0.21)	52.6 (48-57.2)	-1.77 (-4.74; 1.20)
BP	57.4 (37.1-57.4)	57.4 (47.3-57.4)	57.4 (37.1-57.4)	-0.85 (-5.69; 3.99)	47.3 (37.1-57.4)	-3.51 (-11.11; 4.09)	47.3 (26.9-57.4)	-3.75 (-13.92; 6.42)
GH	29.6 (29.6-44.7)	29.6 (29.6-44.7)	29.6 (29.6-44.7)	-2.15 (-6.06; 1.76)	29.6 (29.6-29.6)	-2.95 (-9.26; 3.36)	29.6 (29.6-29.6)	-5.5 (-13.47; 2.48)
VT	67.9 (37.7-67.9)	67.9 (37.7-67.9)	57.8 (37.7-67.9)	-1.18 (-6.62; 4.27)	52.8 (37.7-67.9)	-0.79 (-9.73; 8.14)	57.8 (37.7-67.9)	0.9 (-10.44; 12.25)
SF	56.6 (46.5-56.6)	56.6 (56.6-56.6)	56.6 (36.4-56.6)^∗^	-6.16^∗^ (-10.08; -2.24)	46.5(36.4-56.6)^∗^	-6.51^∗^ (-12.36; -0.66)	56.6 (46.5-56.6)	-5.35 (-14.07; 3.38)
RE	56.1 (50.5-56.1)	56.1 (56.1-56.1)	56.1 (50.5-56.1)	-2.24^∗^ (-3.74; -0.74)	53.3 (44.9-56.1)^∗^	-2.99^∗^ (-5.19; -0.80)	56.1 (56.1-56.1)	0.79 (-2.41; 3.99)
MH	58.4 (46.3-64.5)	58.4 (46.3-64.5)	58.4 (46.3-64.5)	-2.68 (-6.74; 1.39)	52.3 (46.3-64.5)	-2.53 (-9.28; 4.21)	61.5 (52.3-64.5)	4.74 (-3.84; 13.31)
PCS-12	45.6 (38.9-50.5)	46.6 (38.9-50.8)	44 (39.7-48.9)	-1.71 (-4.85; 1.44)	41.7 (34.3-50.1)	-2.96 (-7.96; 2.04)	37.3 (33-43.8)	-3.39 (-10.04; 3.26)
MCS-12	56.3 (51.1-62.3)	58.6 (52.1-62.9)	54.7 (49.8-59.3)^∗^	-3.10^∗^ (-5.85; -0.35)	53 (46.7-60.4)	-2.83 (-7.31; 1.64)	57.8 (53.2-61)	1.56 (-4.36; 7.49)

Figures expressed as median (IQR). BP: bodily pain; GH: general health; MCS: mental component summary score; MH: mental health; PCS: physical component summary score; PF: physical functioning; RE: role emotional; RP: role physical; SF: social functioning; VT: vitality. ^∗^*p* < 0.05. ^a^Multivariate linear regression adjusted for the number of comorbidities, age, sex, duration of disease, HbA1c, glucose, SBP, and DBP. ^b^Compared to without complication.

**Table 4:** Health utility measured by the EQ-5D index according to different diabetic complications.

Diabetic complications	EQ-5D index		
	*n*	Median (IQR)	Difference (95% CI)^a^
Total	214	0.94 (0.85-1.00)	
Number of diabetic complications			
Without diabetic complications	131	1.00 (0.88-1.00)	REF
Having at least one diabetic complication	83	0.94 (0.84-1.00)^∗^	-0.10 (-0.18; -0.01)
Having one diabetic complication	67	0.92 (0.82-1.00)^∗^	-0.10 (-0.20; -0.01)
Having two or more diabetic complications	16	1.00 (0.92-1.00)	-0.06 (-0.25; 0.12)
Complications			
Having diabetic heart disease	56	0.94 (0.86-1.00)	-0.05 (-0.14; 0.04)
Having diabetic nephropathy	18	0.93 (0.85-.100)	-0.08 (-0.23; 0.07)
Having diabetic retinopathy	10	0.92 (0.81-1.00)	-0.17 (-0.37; 0.03)

^∗^ *p* < 0.05. ^a^Multivariate Tobit regression adjusted for the number of comorbidities, age, sex, duration of disease, HbA1c, glucose, SBP, and DBP.

## Data Availability

The data used to support the findings of this study were supplied by the Traditional Medicine Hospital of the Ministry of Public Security under license and so cannot be made freely available. Requests for access to these data should be made to Tam Thi Ngo (ngothitam.tlu@gmail.com).
